# Insulin and insulin-like growth factors act as renal cell cancer intratumoral regulators

**DOI:** 10.1007/s12079-019-00512-y

**Published:** 2019-03-30

**Authors:** Wojciech Solarek, Michal Koper, Slawomir Lewicki, Cezary Szczylik, Anna M. Czarnecka

**Affiliations:** 1 Department of Oncology with Laboratory of Molecular Oncology, Military Institute of Medicine, Szaserow 128, Warsaw, 04-141 Poland; 2 School of Molecular Medicine, Medical University of Warsaw, Warsaw, Poland; 3 Institute of Genetics and Biotechnology, Faculty of Biology, University of Warsaw, Warsaw, Poland; 4 Department of Regenerative Medicine and Cell Biology, Military Institute of Hygiene and Epidemiology, Warsaw, Poland; 5 Department of Oncology, European Health Centre, Otwock, Poland; 6 Medical Center for Postgraduate Education, Warsaw, Poland

**Keywords:** Renal cell carcinoma, Insulin, Insulin-like growth factor, Insulin-like growth factor receptor, Insulin receptor

## Abstract

The risk of renal cell carcinoma development is correlated with obesity and type II diabetes. Since insulin and insulin-like growth factors play a key role during development of both metabolic diseases, these molecules may be important in RCC pathophysiology We investigated the effect of insulin and IGFs on RCC cells using in vitro model with 786-O, 769-P, Caki-1, Caki-2, ACHN cancer cell lines. Cancer cells were compared with normal kidney cells - PCS-400-010 and HEK293. The growth, viability of cells as well as migration rate were assessed upon hormonal stimulation. The insulin receptor and Insulin-like growth factor 1 receptor presence were evaluated and the expression of 84 genes related to insulin signaling pathway. In all RCC cell lines IGF-1R expression was confirmed in contrast to IR, which was expressed only in control HEK293 cell line. Insulin and IGFs stimulated RCC cells growth and migration rate. Insulin, IGF-1 and IGF-2 triggered both IR and IGF-1R phosphorylation. Analyzed RCC did not secret insulin, IGF-1 or IGF-2 and were not activated in autocrine-paracrine signaling loop. Insulin and IGFs stimulations triggered down-regulation of PI3K-Akt-mTOR and Ras-MAPK pathway gens, as well as DOK2–3, INS, FRS3, IRS1–2, IGF1R – genes encoding insulin receptor-associated proteins. In conclusion, we showed that IGFs and insulin may play a stimulatory role for renal cancer cells, thus they can possibly affect renal cancer tumorigenesis and progression on cellular level.

**Electronic supplementary material:**

The online version of this article (10.1007/s12079-019-00512-y) contains supplementary material, which is available to authorized users.

## Introduction

Kidney cancer with about 403,262 new cases diagnosed and 175,098 deaths worldwide is the 16th most common cancer (Bray et al. 2018). With renal pelvis cancer, RCC was predicted to be diagnosed in 63,340 new cases and to cause 14,970 deaths in United States in 2018 (Siegel et al. 2016). It is well known, that diagnosis of obesity and/or type II diabetes mellitus is correlated with the increased risk of RCC development (Murai and Oya 2004; Larsson and Wolk 2011). These diseases are related to deregulation of insulin (IN) and insulin-like growth factors (IGFs) signaling, which are also expected to influence RCC tumorigenesis and progression (Solarek et al. 2015; Labochka et al. 2016; Tracz et al. 2016).

Insulin is produced in the pancreatic islet β cells as a pre-pro-hormone from *INS* gene located in 11p15.5, then gradually cleaved to form active peptide. IN regulates carbohydrate and fat metabolism on cellular and organismal level. Insulin activity is exerted via the Insulin Receptor (IR). IR is mainly expressed in adipose tissue, muscle and liver cells (Matyszewski et al. 2015a, b, c). Interestingly, high serum insulin concentration inhibits autophagocytosis, proteasome activity and apoptosis, which may lead to the antiapoptotic and mitogenic effects (Reuveni et al. 2013; Matyszewski et al. 2015a, b, c). On the other hand IR expression on RCC tumor cells is inversely associated with tumor stage or presence of distant metastases. At the same time hyperinsulinemia was also reported not to enhance tumor growth in murine RENCA RCC animal model (Solarek et al. 2015; Takahashi et al. 2017).

IGFs are produced mainly in liver under the control of growth hormone and in turn regulate cells growth and proliferation. Ligand binding with IGF1R (or IR) leads to activation of tyrosine kinase signaling and phosphorylation of insulin receptor substrate proteins (IRS). Activated IRS in turn induce two crucial intracellular signaling pathways: PI3K-Akt-mTOR pathway and Ras-MAPK pathways that regulate cell proliferation, apoptosis and potentially cancer development (Pollak 2008). In most RCC cases of VHL protein inactivation is found and it was proven that in turn this leads to uncontrolled stimulation of IGF1R-mediated signaling pathway promoting RCC invasiveness through the interaction with RACK1 and subsequent Akt and MMP-2 activation (Datta et al. 2000). It is highly probable that deregulated IR and IGF1R signaling promote development of several cancers but the activity and function of this pathway has not been coherently studied in RCC. The role if insulin and IGFs in RCC pathophysiology has been elusive until now. It may be hypothesized that hyperinsulinemia enhance cancer cells growth and proliferation through insulin’s effect on its cognate receptor, and also by the IGFs pathway activation (Frasca et al. 2008; Solarek et al. 2015).

The aim of the study was to verify the hypothesis that insulin and insulin-like growth factors stimulate renal cancer cells proliferation and viability excessively in comparison to normal kidney cells. We aimed to verify the presence of insulin and insulin-like growth-factor autocrine-paracrine signaling loop in RCC cells and to describe subsequent activation of insulin-related signaling pathway. The ultimate goal of the study was to assess the role of insulin and insulin-like growth factors in the proliferation, growth and migration of primary and metastatic tumor derived renal cancer cells.

## Materials and methods

### Routine cell culture

The renal cancer cell lines 786-O (CRL-™), 769-P (CRL-1933™), Caki-1 (HTB46™), Caki-2 (HTB-47™), ACHN (CRL1611™) and control cell lines PCS-400-010 and HEK293 (CRL 1573™) were obtained from American Type Culture Collection (ATCC) Bioresource Center (Manassas, VA, USA). The characteristics of each cell line are presented in Table 1. The 786-O, 769-P, Caki-1, Caki-2, ACHN cell lines were routinely cultured in RPMI-1640 with GlutaMAX™ Supplement medium (Life Technologies, CA, USA) supplemented with 10% fetal bovine serum (FBS; Biochrom GmbH, Cambridge, UK) and 1% antibiotic solution (penicillin– streptomycin; Invitrogen, CA, USA), in a 37 °C humidified atmosphere with 5% CO2.

**Table 1:** Investigated cell lines

Cell line	Origin	Histopathology
786-O (CRL-1932™)	Primary tumor	Primary Clear Cell Carcinoma (RCC)
769-P (CRL-1933™)	Primary tumor	Primary Clear Cell Carcinoma (RCC)
Caki-1 (HTB46™)	Metastatic tumor	Kidney Carcinoma Metastasis to Skin
Caki-2 (HTB-47™)	Primary tumor	Primary Clear Cell Carcinoma (RCC)
ACHN (CRL1611™)	Metastatic tumor	From the Malignant Pleural Effusion (RCC)
HEK293 (CRL 1573™)	Embryonic kidney	Human Embryonic Kidney Cells
PCS-400-010	Healthy kidney	Primary Renal Proximal Tubule Epithelial Cells

All cell lines were screened for Mycoplasma contamination on a regular basis with Mycoplasma Detection Kit (Jena Bioscience, PP-401 L) and only negative passages were used for the presented experiments. All cell lines passages were not higher than 15. Cell lines were authenticated with ATCC service with short-tandem repeat profiling (STR Profiling Cell Authentication Service, LGC Standards, Lomianki, Poland).

### Experimental cell culture (hormonal stimulation cell culture)

Renal cancer cells or control cells were cultured under standard conditions in cell incubator (37 °C, 5% CO_2_). Cells were moved to 6/24/96 wells plates or T25 flasks. In order to observe influence of insulin and IGFs on cancer cells we used serum free conditions. After 24 h cells were rinse with PBS twice and RPMI 1640 medium with 0,1% BSA (Sigma-Aldrich, USA) was used (serum starved conditions). After next 24 h RPMI 1640 medium with 0,1% BSA and investigated growth factors and/or inhibitors: Insulin (Human Recombinant Zinc, Gibco by Invitrogen, CA, USA), IGF1 (Recombinant Human Insulin Like Growth Factor-1), IGF2 (Recombinant Human Insulin Like Growth Factor-2) (each 100 ng/ml), IR inhibitor - HNMPA-(AM) (100 μM), IGF1R Inhibitor – PPP (50 nM) (Merck Millipore, Darmstadt, Germany) was used for culture.

### Measurement of cell viability

Cells were seeded in 96 well plates (10^4^ cells/ml) and cultured under standard conditions (37 °C, 5% CO_2_) according to the experimental protocol. Viability was measured in 72 h and 96 h with AlamarBlue(resazurin) (Thermo Fisher Scientific, MA, USA) assay according to manufacturer’s protocol. Absorbance was read by microplate reader Multiskan GO and analyzed using ScanIt™ software package (Thermo Fisher Scientific, MA, USA). Growth curves were acquired with cells seeded (10^4^ cells/ml) in T25 flasks and cultured under standard conditions according to the experimental protocol. Each day of experiment from one of T25, cells were harvested by trypsynization (0.25% trypsin, 0.03% EDTA solution, Life Technologies, Carlsbad, CA, USA) and counted in an automated cell counter (MOXI Z, Orflo Technologies, Ketchum, ID, USA). All experiments have been conducted in at least 3 replicates.

### Wound-healing assay

Cells were seeded in 24-well plates and cultured according to the routine protocol. Cell migration was assessed by the wound-healing assay (Grada et al. 2017). Cells were rinse with PBS twice and RPMI with 0,1% BSA medium was added (serum starved conditions). After 24 h RPMI with 0,1% BSA medium with investigated factors and inhibitors: Insulin, IGF1, IGF2 (each 100 ng/ml) was added and a wound was introduced in a cell monolayer using a pipette tip (at least 95% of confluence). The ‘wounded’ areas were photographed at 0, 24, 48, 72, 96 h. The relative migration distances were analyzed using Image J Software (version 1.41; National Institutes of Health). All experiments have been conducted in at least 2 replicates.

### FACS

Cells were seeded in 6 well plates and cultured under standard conditions (37 °C, 5% CO_2_) according to the routine protocol. Cells were washed twice in PBS and detached by Trypsin/EDTA for 5 min at 37 °C. The cells were subsequently washed once in PBS and incubated with 5 μl sample of PE conjugated mAb anti-IGF1R (CD221), anti-IR (CD220) or mouse IgG κ isotype control (Becton Dickinson, NJ, USA) for 30 min at 4 °C in PBS. The cells were washed twice in PBS to remove unbound antibody, resuspended in PBS-buffered formalin and then analyzed using a FACS flow cytometer (FACSCalibur™, Becton Dickinson, NJ, USA) according to standard settings at 488 nm. Each sample was prepared in duplicate and analyzed for IR and IGF1R expression.

### Multiplex analysis

Cells were seeded in 6 well plates and cultured under standard conditions (37 °C, 5% CO^2^) according to the experimental protocol (serum starved cells). 10 min after the addition of insulin, IGF-1, or IGF-2 cells were washed twice with ice-cold PBS, lysed with ice-cold Lysis Buffer containing Halt Protease and Phosphatase Inhibitor (Thermo Fisher Scientific, MA, USA). Total protein concentration was measured with the BCA Protein Assay (Thermo Fisher Scientific, MA, USA). Total and phosphorylated IGF1R and IR were detected with MILLIPLEX MAP Total/Phospho Mitogenesis RTK Magnetic Bead 7-Plex Kit (Merck Millipore, Darmstadt, Germany). Alternatively after 24 h cells supernatant was collected and Insulin, IGF1 and IGF2 concentrations were measured using Human Insulin ELISA for insulin and MILLIPLEX MAP Human IGF-I, II Magnetic Bead Panel (Merck Millipore, Darmstadt, Germany). The results were shown as a MFI – mean fluorescent intensity of three repeats for each sample.

### Elisa

Cells were seeded in 6 well plates and cultured under standard conditions (37 °C, 5% CO^2^) according to the routine cell culture protocol. For IR expression measurement cells were washed twice with cold PBS, lysed with ice-cold lysis RIPA buffer (Sigma-Aldrich, MO, USA) containing Halt Protease and Phosphatase Inhibitor (Thermo Fisher Scientific, MA, USA). Total protein concentration was measured with the BCA Protein Assay (Thermo Fisher Scientific, MA, USA). IR concentration was measured with STAR IR (β subunit) ELISA Kit (Merck Millipore, Darmstadt, Germany).

### RT-PCR

Cells were seeded in T25 flasks and cultured under standard conditions (37 °C, 5% CO^2^) according to the experimental protocol. After 24 h total RNA samples from three independent biological replicates were isolated using miRNeasy Mini Kit (Qiagen, CA, USA), according to the manufacturer’s instructions. RNA quality and concentration were measured using RNA Nano chip on the 2100 Bioanalyzer instrument (Agilent Technologies) and only RNA samples with RIN value (RNA Integrity Numbers) not lower than 9.0 were selected for reverse transcription. cDNA was synthesized from 400 ng of RNA using the RT2 First Strand Kit (Qiagen, CA, USA), according to the manufacturer’s instructions. RT-qPCR was performed in triplicate, using one plate for one experiment repetition (384-well plates divided in to 96-well parts for control and each investigated condition). To each well of 384-well plates (RT^2^ Profiler™ PCR Array Human Insulin Signaling Pathway) we added 10-μl reaction mixture containing 5 μl of 2x RT^2^ SYBER Green Mastermix (Qiagen, CA, USA) and 5 μl of cDNA synthesis reaction with RNase-free water. Reactions were pipetted using JANUS® Extended Integrator 8-tip Automated Workstation (PerkinElmer). C*p* values were calculated using LightCycler®480 Software 1.5 (Roche Diagnostics), based on the Second Derivative Maximum Method. CT values were exported to an Excel file to create a table of CT values. This table was then uploaded on to the data analysis web portal at http://www.qiagen.com/geneglobe. Samples were assigned to controls and test groups. CT values were normalized based on a/an Automatic selection from full panel of reference genes. The data analysis web portal calculates fold change/regulation using delta delta CT method, in which delta CT is calculated between gene of interest (GOI) and an average of reference genes (HKG), followed by delta-delta CT calculations (delta CT (Test Group)-delta CT (Control Group)). Fold Change is then calculated using 2^ (−delta delta CT) formula.

### Statistical analyses

The data were analyzed as the means with standard deviation (SD) of at least three experiments. Data fitting and statistical analysis were performed and graphs were prepared using Microsoft’s Excel program 2013 (Washington, USA) and Biovinci web version (https://vinci.bioturing.com). Statistical analysis was performed using ANOVA followed by Tukey’s HSD test as statistical methods that allow estimation of inter-individual variability in intra-individual patterns of change over time. Differences and relationship were considered statistically significant when *P* = 0.05. Gene expression level (RT-qPCR) was also evaluated with t-test .

## Results

### RCC cells express IGF1R, but not IR

**Fig. 1:**
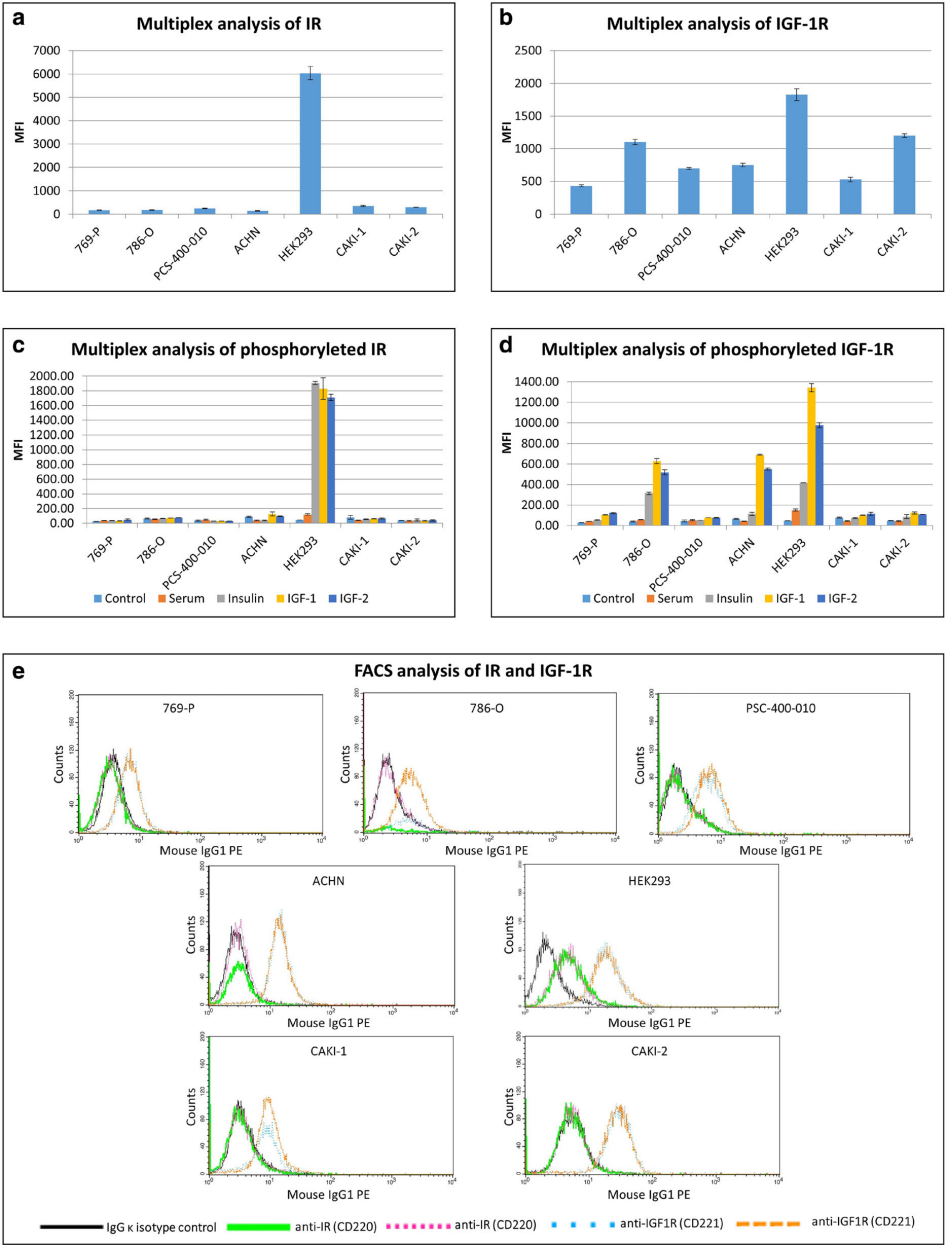
**Total and phosphorylated IR and IGF1R analysis.** The only HEK293 cell line express IR receptor. It is present in cell lysate what was assessed with multiplex Luminex analysis (relative quantity shown as MFI – mean fluorescent intensity of three repeats for each sample) (**a**). The expression of IR was also observed on the cell surface in FACS analysis with PE conjugated mAb anti-IR (CD220). Green and pink curves represents two repeats of IR analysis, blue and orange represents IGF-1R analysis and black stays for isotype control (**e**). These results were also confirmed with ELISA assay. Treatment with each investigated molecule (insulin, IGF1, IGF2) lead to IR phosphorylation (**c**). Both cancer cell line and control cell line expressed IGF1R visible on cell surface (**e**) and in cell lysate (**b**). IGF1R phosphorylation was observed after insulin, IGF1 and IGF2, what was most strongly pronounced in cell lines 786-O, ACHN and HEK293 (**d**)

Among all investigated RCC cell lines, including primary tumor derived and metastasis derived cell lines as well as normal cell lines, IGF1R expression was confirmed with FACS (Fig. 1e, Suppl. 1). ACHN, Caki-2 and HEK293 cell lines presented the highest number of expression-positive cells, which was more than 90%. The presence of IGF1R was also confirmed with multiplex Luminex analysis using cells lysates (Fig. 1b, d). In this analysis the highest expression was detected in ACHN, HEK293 and 786-O cell lines. IR surface expression was found only in HEK293 embryonic kidney cell line. Only 11% of HEK293 cells expressed this receptor (Fig. 1e – FACS). The result was confirmed in cell lysate using two another methods – ELISA assay (data not shown) and multiplex Luminex analysis (Fig. 1a, c). In ELISA assay the concentration of β subunit of insulin receptor was only 2,77 ng/ml in this kidney cell line. In all RCC cell lines and adult renal proximal tubule epithelial cells no expression of IR β subunit protein was found. This result was confirmed with in multiplex magnetic analysis. The mean fluorescence intensity (MFI) for IR in HEK293 cell line was 6036.19 a.u. and neglectable (below 300 a.u.) for all other investigated cell lines. Using all three methods we show the lack of IR receptor expression on RCC cell lines, and/or IR accumulation within RCC cells, despite the fact that those cell lines are responsive to insulin stimulation (as described below).

### Insulin and IGFs stimulate RCC cells proliferation and migration

Both primary tumor derived, and metastatic tumor derived RCC cells, as well as embryonic kidney cells (HEK-293), were found to be fast proliferators in contrast to adult renal proximal tubule epithelial cells (PCS-400-010). PCS-400-010 present slow proliferation rate, also in hormone-supplemented medium with Insulin 5 μg/ml (and Triiodothyronine 10 nM, rh EGF 10 ng/mL, Hydrocortisone 100 ng/mL, Epinephrine 1.0 μM). For all renal cells serum free conditions limit the exponential growth phase. Most of cell lines are able to survive serum restriction for 3 to 4 days and become necrotic at day 5th upon serum starvation.

**Fig. 2:**
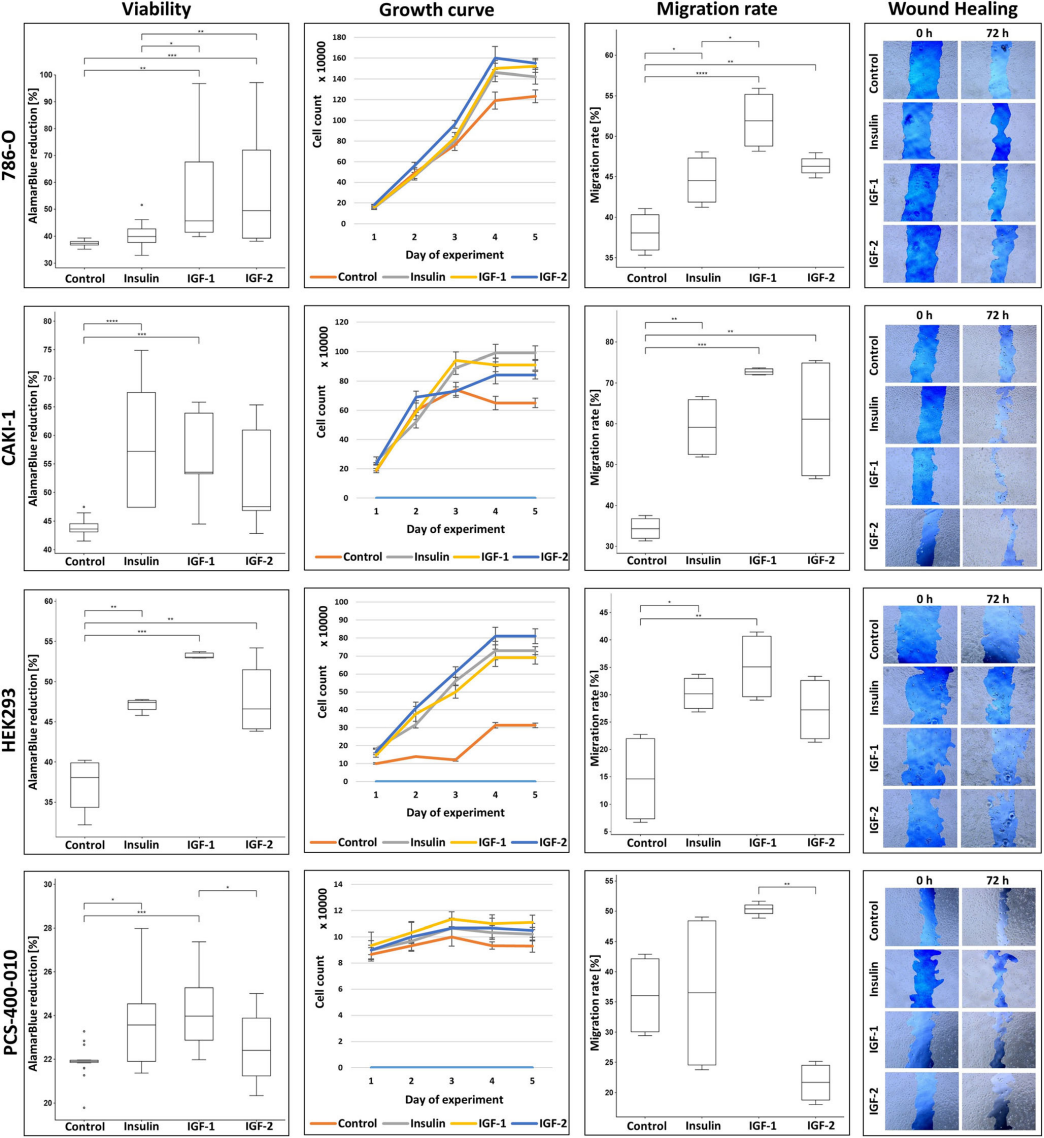
Cells viability and migration rate after insulin, IGF1 or IGF2 stimulation. First column present cells viability measured in AlamarBlue test. Median (line) and interquartile range (box) are shown along with minimum and maximum data values. In this assay resazurin upon entering living cells is reduced to resorufin, which can be quantify by absorbance reading (Y-axis). Presented results were acquired 72 h after addition of insulin, IGF1 or IGF2. The second column show cells growth curves, starting from 1st day of experiment (24 h after addition of investigated compounds) till day 6th when cells reached plateau phase of growth. Third column visualize migration rate of investigated cell lines after addition of insulin, IGF1 or IGF2 in 72 h (786-O, CAKI-1, HEK293) or 96 h (PCS-400-010 due to slow proliferation rate) in wound healing assay. The migration rate [%] was calculated as a percentage decrease in wound area between start and 48 or 96 h of experiment, calculated with Image J software (Y-axis). Fourth column represent wound healing assay visualization with enhanced contrast (Image J) in wound area in the beginning and 72 or 96 h of experiment. Statistical analysis was performed using ANOVA followed by Tukey’s HSD (Biovinci web version - https://vinci.bioturing.com). * *P* ≤ 0.05, ** *P* ≤ 0.01, *** *P* ≤ 0.001, **** *P* ≤ 0.0001

**Fig. 3:**
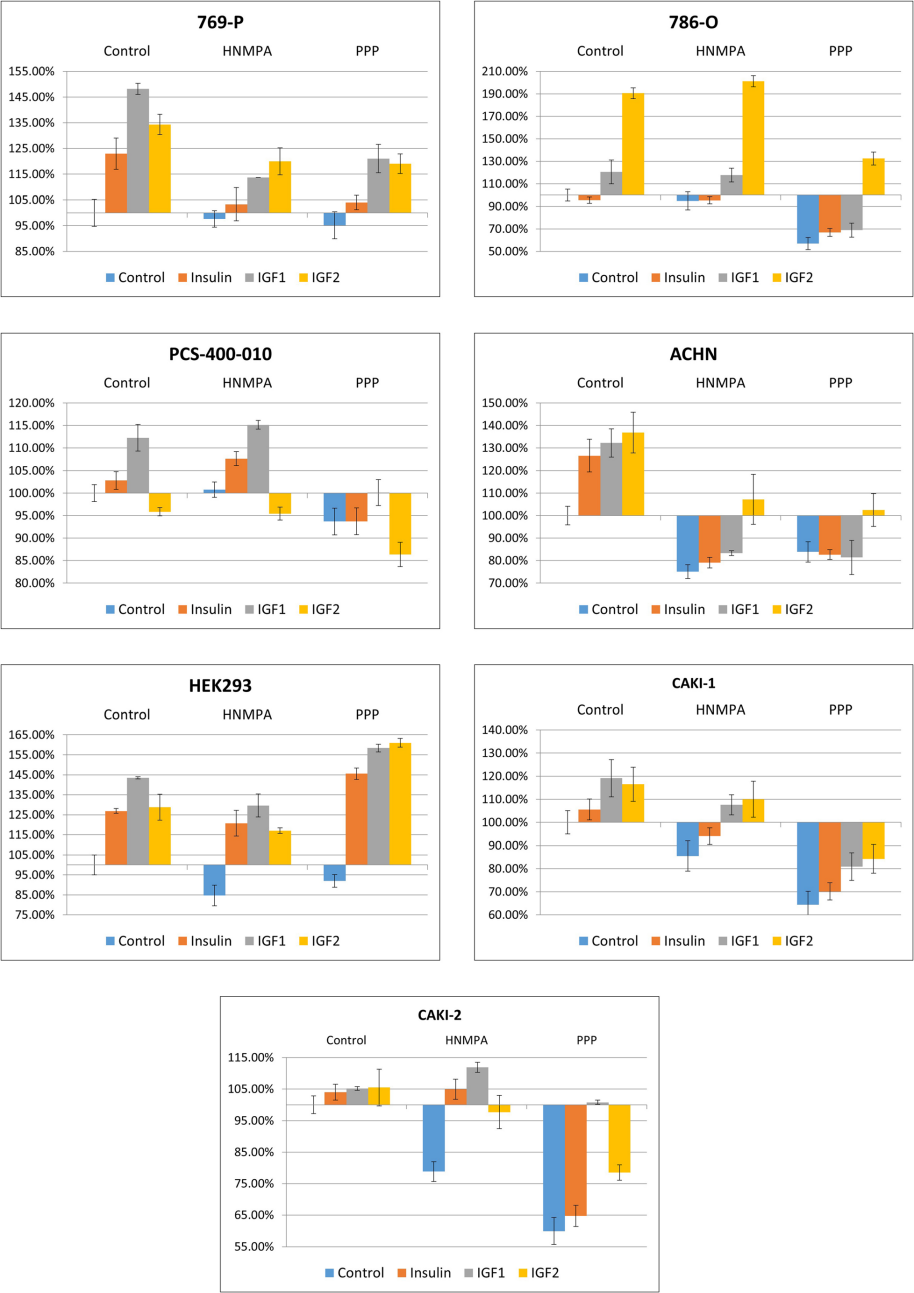
**The influence of IR and IGF1R inhibitors on cells viability**. The cells viability was assessed using Alamar blue test in 72th hour of experiment (all investigated factors and inhibitors were added at beginning of experiment). Insulin receptor inhibition with HNMPA-(AM) (not specific inhibition) decreased the viability in all tested conditions. PPP (IGF1R inhibitor) dismiss the effect of insulin. After IGF1 or IGF2 there was no specific differences in the viability of cells cultured with HNMPA-(AM) nor PPP

Both metastatic cell lines (Caki-1, ACHN) and control normal renal embryonic and adult renal cell lines (HEK293 and PCS-400-010) were significantly proliferation-responsive to stimulation with insulin (Fig. 2.), with most profound effect on embryonic cells (HEK293) and metastatic RCC cells (Caki-1). Addition of insulin to primary tumor derived RCC cell lines (Caki-2, 769-P, 786-O) did not affect it viability or proliferation in first 72 h after stimulation. Insulin receptor inhibition with Hydroxy-2-naphthalenylmethylphosphonic Acid Trisacetoxymethyl Ester (HNMPA-(AM)) - Insulin receptor tyrosine kinase inhibitor - did not result with specific insulin-only stimulation inhibition (Fig. 3., Suppl. 2). Inhibition of IR tyrosine kinase signaling as well as serine and tyrosine autophosphorylation decreases the viability of RCC cells in all tested conditions, including insulin (and IGFs) stimulation. In fact the decreased viability of RCC cells in growth factor restriction conditions with concurrent HNMPA-(AM) treatment is expected to result from decreased insulin-stimulated glucose oxidation. Finally analyzed RCC were found not to secret insulin, IGF1 or IGF2 in autocrine manner. In all tested supernatants no Insulin, IGF1 nor IGF2 was detected in (serum-free) culture (Tables 2, 3 and 4).

**Table 2:** Concentration of insulin

	Control	Insulin	IGF1	IGF2	HNMPA	PPP
769-P	<0,04 ng/ml	<0,04 ng/ml	<0,04 ng/ml	<0,04 ng/ml	<0,04 ng/ml	<0,04 ng/ml
786-O	<0,04 ng/ml	<0,04 ng/ml	<0,04 ng/ml	<0,04 ng/ml	<0,04 ng/ml	<0,04 ng/ml
PCS-400-010	<0,04 ng/ml	<0,04 ng/ml	<0,04 ng/ml	<0,04 ng/ml	<0,04 ng/ml	<0,04 ng/ml
ACHN	<0,04 ng/ml	<0,04 ng/ml	<0,04 ng/ml	<0,04 ng/ml	<0,04 ng/ml	<0,04 ng/ml
HEK293	<0,04 ng/ml	<0,04 ng/ml	<0,04 ng/ml	<0,04 ng/ml	<0,04 ng/ml	<0,04 ng/ml
CAKI-1	<0,04 ng/ml	<0,04 ng/ml	<0,04 ng/ml	<0,04 ng/ml	<0,04 ng/ml	<0,04 ng/ml
CAKI-2	<0,04 ng/ml	<0,04 ng/ml	<0,04 ng/ml	<0,04 ng/ml	<0,04 ng/ml	<0,04 ng/ml

**Table 3:** Concentration of IGF1

	Control	Insulin	IGF1	IGF2	HNMPA	PPP
769-P	<41 pg/ml	<41 pg/ml	81 pg/ml	<41 pg/ml	<41 pg/ml	<41 pg/ml
786-O	<41 pg/ml	<41 pg/ml	<41 pg/ml	<41 pg/ml	<41 pg/ml	<41 pg/ml
PCS-400-010	<41 pg/ml	<41 pg/ml	<41 pg/ml	<41 pg/ml	<41 pg/ml	<41 pg/ml
ACHN	<41 pg/ml	<41 pg/ml	<41 pg/ml	<41 pg/ml	<41 pg/ml	<41 pg/ml
HEK293	<41 pg/ml	<41 pg/ml	81 pg/ml	<41 pg/ml	<41 pg/ml	<41 pg/ml
CAKI-1	<41 pg/ml	<41 pg/ml	115 pg/ml	<41 pg/ml	<41 pg/ml	<41 pg/ml
CAKI-2	<41 pg/ml	<41 pg/ml	<41 pg/ml	<41 pg/ml	<41 pg/ml	<41 pg/ml

**Table 4:** Concentration of IGF2

	Control	Insulin	IGF1	IGF2	HNMPA	PPP
769-P	<0,9 ng/ml	<0,9 ng/ml	<0,9 ng/ml	4,2 ng/ml	<0,9 ng/ml	<0,9 ng/ml
786-O	<0,9 ng/ml	<0,9 ng/ml	<0,9 ng/ml	<0,9 ng/ml	<0,9 ng/ml	<0,9 ng/ml
PCS-400-010	<0,9 ng/ml	<0,9 ng/ml	<0,9 ng/ml	<0,9 ng/ml	<0,9 ng/ml	<0,9 ng/ml
ACHN	<0,9 ng/ml	<0,9 ng/ml	<0,9 ng/ml	<0,9 ng/ml	<0,9 ng/ml	<0,9 ng/ml
HEK293	<0,9 ng/ml	<0,9 ng/ml	<0,9 ng/ml	8,5 ng/ml	<0,9 ng/ml	<0,9 ng/ml
CAKI-1	<0,9 ng/ml	<0,9 ng/ml	<0,9 ng/ml	<0,9 ng/ml	<0,9 ng/ml	<0,9 ng/ml
CAKI-2	<0,9 ng/ml	<0,9 ng/ml	<0,9 ng/ml	<0,9 ng/ml	<0,9 ng/ml	<0,9 ng/ml

On the contrary to delayed proliferation effect, significant insulin influence on migration and proliferation rate of primary tumor derived RCC cells’ was observed in wound healing assay over 3 days (Fig. 2.). Concordantly to the primary tumor derived RCC cells’ insulin rapidly increased migration rate of all metastatic cancer cell lines and embryonic kidney cell line. The increased potential of wound healing was significant in metastatic cell lines (*p* < 0.005 vs *p* < 0.05 for primary cancer site cell lines), which suggest that insulin conduct its stimulating signal through the IGF1R receptor in this case.

All cell lines except Caki-2 were strongly responsive to stimulation with IGF1. Both the viability and migration rate where significantly increased after IGF1 stimulation. This effect was most prominent on day 3rd and 4th of stimulation. In Caki-2 cells response to IGF1 stimulation was insignificant. The increased viability was more profound in metastatic RCC cell lines than in primary cancer derived RCC cell lines (p < 0.005 vs p < 0.05 respectively). Exposure to IGF2 stimulated viability and migration rate of all cell lines except primary normal kidney cell line, which was not responsive to this stimulation. Viability (cellular metabolism) of Caki-1 cell line after IGF2 stimulation was not significantly increased (AlamarBlue) but proliferation (growth curve) and migration (wound healing assay) were augmented (Fig. 2.). Renal cells stimulation by IGF1 or IGF2 was inhibited with HNMPA-(AM) nor PPP. Both IGF inhibitors decreased cell viability irrespectively of stimulating growth factor.

#### Both IR and IGF1R are phosphorylated after IGF1, IGF2 and insulin stimulation

The phosphorylation of IR(panTyr) and IGF1R (panTyr) was assessed in cell lysate with phosphatases and phosphorylases inhibitors (Fig. 1. C, D - Luminex assay). The most significant phosphorylation of IGF1R after IGF1 stimulation was observed in 786-O, ACHN and HEK293 cells. IGF2 stimulation was less effective, but also most profound in 786-O, ACHN and HEK293 cells. Interestingly, the stimulation with IN resulted in phosphorylation of IGF1R in 786-O, Caki-2 and HEK293 cell lines. The insulin receptor that is expressed only in HEK293 cells, was phosphorylated upon its ligand (IN) binding. Also IGF1 and IGF2 induced IR phosphorylation in these cells (Fig. 1c, d).

#### Insulin and IGFs signaling pathway is affected after insulin, IGF1 and IGF2 stimulation

**Fig. 4:**
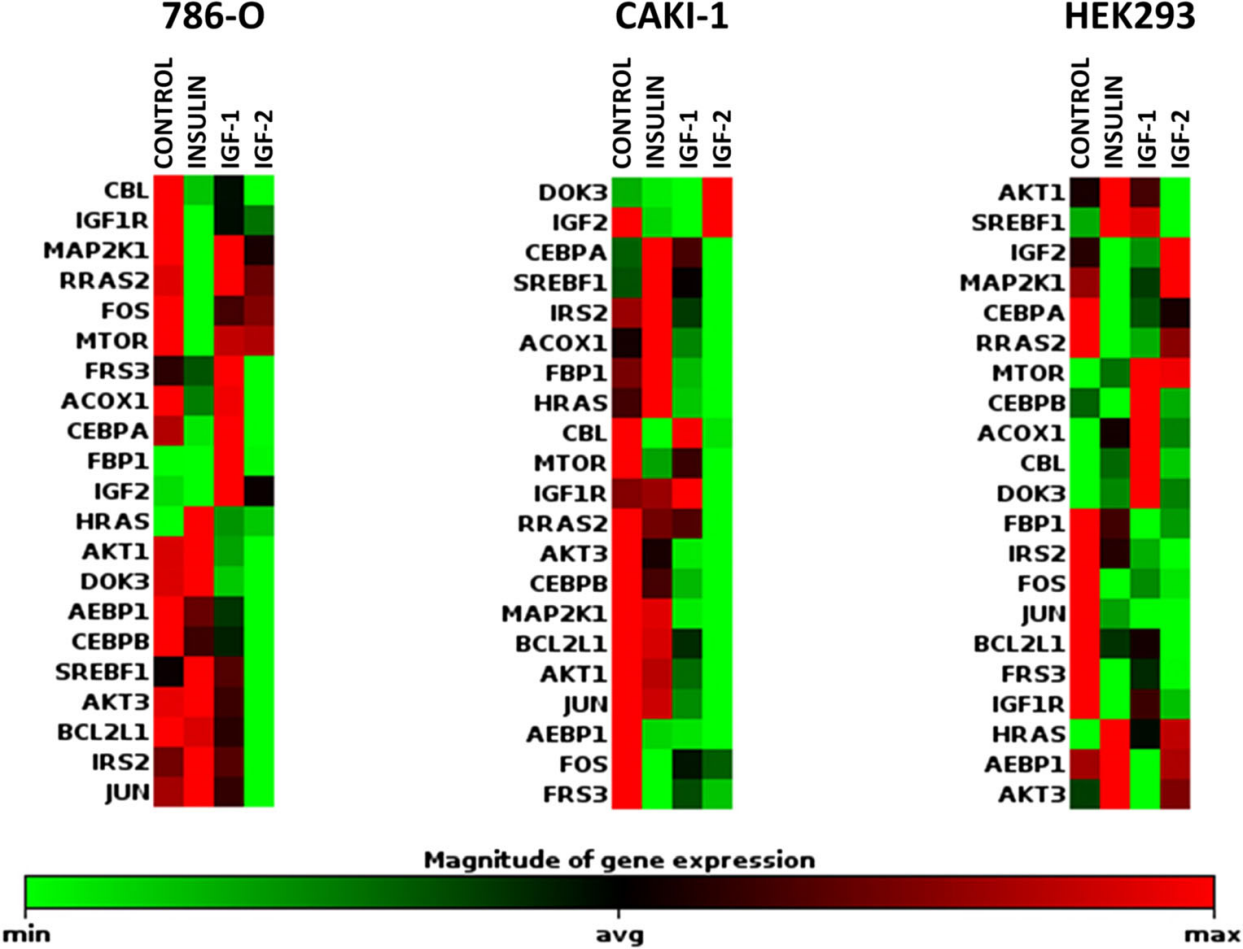
**Human Insulin Signaling Pathway gene expression analysis.** Among 84 genes which were assessed with RT^2^ Profiler™ PCR Array Human Insulin Signaling Pathway (Qiagen) the change in expression was revealed in expression of genes related to insulin-associated proteins (DOK1–3, INS, FRS3, IRS1–2, IGF1R), transcription regulators (AEBP1), PI3 kinase signaling (AKT1, BCL2L1, MTOR), MAP Kinase Signaling (FOS, MAP2K1, HRAS, RRAS) as well as carbohydrate (FBP1) and lipid metabolism (ACOX1)

The insulin stimulation lead to significant down-regulation of genes related to MAP Kinase Signaling - such as *FOS* and *MAP2K1* - expression in 786-O cells or *RRAS2* gene in HEK293 cell line (Table 5, Fig. 4). No statistically significant influence on preselected gene expression was found in Caki-1 cell line after insulin stimulation. In HEK293 cell line we also observed decreased expression of insulin receptor-associated proteins genes including *FRS3, IGF1R* or *IGF2*. Insulin stimulation resulted in minor increase in *PRL* gene expression in Caki-1 and HEK293 cell lines.

**Table 5:**
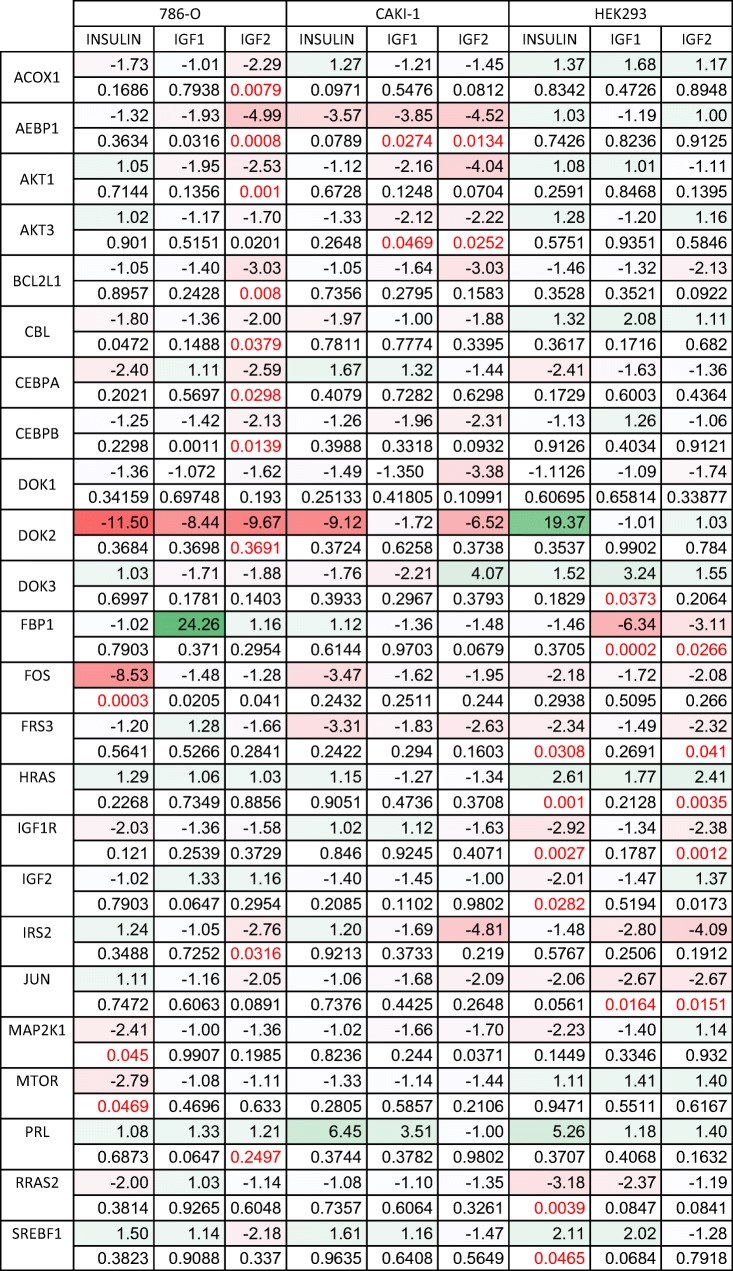
Human Insulin Signaling Pathway gene expression analysis. Fold change with p value below

The fold change/regulation were calculated using delta delta CT method. In which delta CT is calculated between gene of interest (GOI) and an average of reference genes (HKG). followed by delta-delta CT calculations (delta CT (Test Group)-delta CT (Control Group)). Fold Change is then calculated using 2^ (−delta delta CT) formula

After IGF1 stimulation we notice decrease in expression of transcription regulator AEBP1 and PI3 Kinase Signaling protein AKT genes in Caki-1 metastatic RCC cell line. The IGF1 induction of HEK293 cell line resulted in increased expression of Downstream of Tyrosine Kinases (*DOK3)* gene, which encode protein associated with insulin receptor. In this cell line we also observed decreased expression of *FBP1,* which is engaged in glucose metabolism and *JUN* transcription factor. We do not detect the significant change in gene expression after IGF1 stimulation in 786-O cell line.

Subsequently to IGF2 stimulation modulated expression of multiple genes in all investigated cell lines. In 786-O primary tumor cell line we observed decrease expression of genes of insulin-associated proteins (*IRS2, CBL)*, transcription regulators (*AEBP1)*, PI3 kinase signaling (*AKT1, BCL2L1)* as well as lipid metabolism genes (*ACOX1*). In metastatic Caki-1 cell line we showed the down-regulation of transcription factor gene *AEBP1* and PI3 kinase signaling component (*AKT3*). IGF2 conditioning of HEK293 cell line leads to increased expression of *HRAS* gene of MAP kinase pathway and decreased expression of insulin-associated proteins (*FRS3*, *IGF1R)* genes, as well as transcription regulator *JUN* gene and carbohydrate metabolism *FBP1* gene.

In general IN and IGF stimulation did not substantially deregulate gene expression in RCC cells, but actually down-regulation of expression was detected in most cases. The lowest relative expression was detected in insulin receptor-associated pathway *DOK1–3, INS, FRS3, IRS1–2, IGF1R*. Interestingly in the cancerous cell lines missing IR receptor - but not in HEK293 cells - insulin, IGF1 or IGF2 stimulation induced substantial down-regulation of the *INS* gene expression. Another difference between RCC and normal kidney cell lines was observed in Downstream of Tyrosine Kinases (*DOK*) genes expression. Every stimulation of cancer cell lines missing IR lead to down-regulation of *DOK* genes, whereas in control cell line with IR insulin or IGF1 stimulation resulted in up-regulation of *DOK* genes expression. What is also interesting we observed increased expression of *PRL* gene after stimulation with insulin or IGF1 in Caki-1 and HEK293 cell lines.

## Discussion

IGFs and insulin system is complex and dependent on three ligands (insulin, IGF1 and IGF2) and at least three receptors IR, IGF1R as well as IGF type 2 receptor (IGF2R), known as a mannose 6-phosphate receptor (M6P-R), which binds IGFs but does not transduce signal (Leboulleux et al. 2001; Zhang et al. 2010). IR and IGF-IR most often are covalently bound receptor dimers and localize on cell membrane. Although IR and IGF-IR function most often as homodimer, nevertheless hybrid receptors of IR and IGF-IR have also been described before. Those hybrid receptors, irrespective of their splice variants have affinity for insulin, IGF-I, and IGF-II binding, but for IGF-I it is at least 50-fold higher affinity than for insulin and binding characteristics of insulin and IGF-I to both hybrid receptors is similar to regular IGF-IR homodimer (Slaaby et al. 2006). Differences in the activation of different intracellular signaling pathways leading to distinct regulations of gene expression by homo- and heterodimers. Studies proved that intracellular domain of the IR is more effective in phosphorylating IRS-1 thus inducting the expression of genes involved in metabolic pathways; whereas, intracellular domain of IGF1R phosphorylate more potently Shc and Gab-1, which leads to the activation of genes involved in proliferation (Cai et al. 2017). As we have shown here IR is not widely distributed on RCC cells surface, but this does not exclude IN stimulation via alternative receptors. We believe that IGFRs and hybrid receptors are responsible for insulin effects on these RCC cells, as it was recently shown for breast cancer cells (Chen et al. 2018).

In healthy individuals IR is expressed in nearly all absorptive epithelial cells along the renal tubule, including proximal tubule epithelial cells, which give origin to RCC (Tiwari et al. 2013). Strong expression of IR is typical in proximal and distal tubules of normal human renal cortex in healthy individuals, while a significant reduction is found in type 2 diabetic patients, insulin-resistant cases and in those with diabetic nephropathy (Gatica et al. 2013). Insulin impact both metabolic and transport functions of the proximal tubule cells, including gluconeogenesis. IRs are localized on the basolateral side of proximal tubule cells bind serum insulin and activate intracellular signaling (Sasaki et al. 2017). IR expression in RCC tumors was recently shown to be inversely associated with disease progression. IR expression in RCC tumors is low in patients with tumor stage pT2 to pT4 and in those with metastatic disease (Takahashi et al. 2017). We have confirmed IR and IGF1R RCC cell surface expression using FACS with PE conjugated mAb anti-IGF1R (CD221) or anti-IR (CD220). Our results confirm down-regulation of IR expression in RCC cancer cells, but confirm the utility of cell culture models for hormonal investigations in the field of RCC. We have previously shown and confirm with this study that analyzed RCC cell lines represent feasible model for endocrine research in urologic oncology including clear cell and papillary RCC, 2D and 3D cultures as well as hormone restriction conditions (Brodaczewska et al. 2016; Czarnecka et al. 2016; Bielecka et al. 2017; Maliszewska-Olejniczak et al. 2018).

In the field of RCC only a few studies investigating IGFs were published and reported conflicting results: varying degrees of IGFIR expression, as well as complete absence of IGF1R and its ligand IGF1 in RCC tissues are known. As in the case of insulin and IR, the expression of the ligand (IGF1) and its receptor (IGF1R) within the same tumor could provide proof of an autocrine-paracrine signaling loop of RCC cells stimulation (Schips et al. 2004). In particular SN12K1 (metastatic RCC) cell line express autocrine IGF1 (Cheung et al. 2004), but this is not the case with RCC cell lines selected by us, therefore we believe this may be case-specific phenomenon, but is not ubiquitous phenomenon in RCC. Our result stay in accordance with pathological reports that show that the expression of IGF1R is not related to the expression of its ligands, neither in papillary nor in clear cell RCC tumors. Therefore, we provide physiological confirmation of the hypothesis that IGFs signaling in RCC is mediated mainly by circulating ligand proteins (IGF1 and IGF2) from sources other than RCC cancer tumor itself, as suggested before (Schips et al. 2004). We believe that significant impact on RCC cancer cells might be exerted by tumor-associated endothelial cells expressing IGF. Such interaction could promote RCC cells chemo-resistance, but also favor cancer stem cell phenotype (Cao et al. 2017; Youssef et al. 2017). More research is needed in the field, but co-culture bio-mimetic models should also be employed as we have indicated before (Bielecka et al. 2017; Kaminska et al. 2017).

Our results stay also in accordance with the fact that in healthy individuals only 25% of IGF1 is synthesized locally in kidney and acts as an autocrine or paracrine factor. On tissue level local IGF1 is generally limited to stromal cells and act on adjacent (epithelial) cells that do not actually express IGF1. IGF1 detected in the proximal epithelial cells is most probably sequestrated from the circulation (Kamenicky et al. 2014). As we have shown it is both IGFs and insulin that stimulate RCC cells viability and proliferation. For HEK-293 cells it was previously defined that insulin, transferrin and specific lipid mix positively affect cell growth in serum-free media formulations, with optimal insulin concentration of 19,8 mg/L as defined in Box-Behnken experimental design analysis. These cells triplicated their maximum cell densities in the presence of FBS 9,3 × 10^6^ cells/ml in comparison to serum restriction, while in the presence of optimal insulin-transferrin-lipid combination/concentrations of supplements cells reached a maximum cell density of 5,4 × 10^6^ cells/mL at day 5 of the culture (Cervera et al. 2011). Our results prove the pro-proliferative effect of insulin and IGFs in exponential phase of cell culture. The timing of primary proliferative effect seem to stay in correlation with RCC cells doubling time, that is 45 h for 786–0 cell line (Williams et al. 1978), and 32 h for ACHN, 36 h for Caki-1 cells, and 24–30 h for HEK293 cells (Cowley et al. 2014). The activating insulin concentration used in our model is within the insulin blood level, as the concentration of insulin is specific for kidney parenchyma and different that general serum concentration, that is in renal venous blood IN concentration is invariably lower than in the corresponding kidney arterial blood - with the mean arterial insulin level of 14.1 μU and the mean venous level 9.3 μU per ml (0.3 μg/ml) while for IN 1 international unit IU = 0.0347 mg (Chamberlain and Stimmler 1967). Moreover the concentration of insulin in the kidney is higher in proximal tubules than in the glomeruli (Aun et al. 1975). Our experimental design was used to support clinical relevance of these basic observations, since we used stimulation with 0.1 μg/ml that is physiologically detectable in the kidney, even though physiological concentration of insulin in humans serum in peripheral circulation is much lower - 0.5-5 ng/ml (Nakamura et al. 2015).

In normal kidney cells insulin has an inhibitory effect on renal gluconeogenesis, it suppress expression and activity of gluconeogenic enzymes, including phosphoenolpyruvate carboxykinase (PEPCK) and G6Pase (Gatica et al. 2013; Tiwari et al. 2013). As a consequence increased renal glucose-6-phosphatase gene expression is found in IR knock-out and enhanced gluconeogenesis is found when insulin is not able to execute its function in kidney cells (Tiwari et al. 2013). This is not true for RCC cells because in RCC gluconeogenic enzyme fructose-1,6-bisphosphatase 1 (FBP1) is depleted in majority of the cases (Li et al. 2014). Therefore IR loss in RCC should be considered as proliferation dependent, but not metabolism – defining event. It may be therefore mechanism resembling vascular smooth muscle cell, that lacking the IR and characterized therefore by loss of canonical insulin signaling exhibit greater proliferation and migration rates compared to wild type cells. This phenomenon is characterized by increased activation of the ERK-1/2 and decreased p27^Kip1^ in response to stimulation with physiological insulin (Lightell Jr. et al. 2011). As we have shown RCC cells compared with normal renal cells also exhibit greater proliferation and migration concomitant with IR loss. Such IR-loss mediated activity stay in opposite to breast cancer, where downregulation of IR inhibits cancer cell proliferation, angiogenesis and lymphangiogenesis (in animal model). More over downregulation of IR inhibits cancer metastasis in an athymic mouse breast cancer model, even in the presence of functional IGF1R (Zhang et al. 2010), but these cells represent different metabolic abnormalities and FBP mediated metabolism deregulation (Hsieh and Cheng 2016).

The lack of IR receptor on RCC cells lines does not abrogate insulin activity on RCC cells, as analyzed cell lines are responsive to insulin stimulation. As we have shown Insulin may influence RCC cells growth through IGF1R receptor stimulation, what is suggested by receptor phosphorylation tests results. Insulin activity on RCC cells is a result of the phenomenon that both receptors - IR and IGFR, as well as their heterodimers - can bind all three ligands (insulin, IGF1I and IGF2), although with varying affinities. In fact IGFs bind the primary binding site of IGF1R in mode equivalent to that of insulin to IR. This is supported by high degree of similarity of receptor-binding surfaces to those of insulin, and IR and IGF1R 50% sequence homology (Cai et al. 2017; Xu et al. 2018). Phosphorylation of IGF1R in 786-O, Caki-2 and HEK293 cell lines is probably the mechanism through which insulin can affect cells activity despite the lack of insulin receptors on RCC cell lines. Also IGF1 and IGF2 induced IR phosphorylation in these cells (Fig. 1c, d). It may potentiate its stimulating strength as IGFs can influence cells growth and proliferation through IGF1R- and IR-dependent mechanisms. At the same time IN stimulation results in minor increase in *PRL* gene expression in Caki-1 and HEK293 cell lines. This data is consistent with the rest of our results the insulin stimulation affects gene expression in cell lines missing insulin receptor, as described before (Siddle 2012).

Although high degree of homology, IR and IGF1R mediate different and distinct biological cellular functions. Biological processes modulated by these two receptors are different despite common intracellular signaling pathways activated. IGF1R induce more activation of Shc and Gab-1 proteins and more potent regulation of genes involved in proliferation, which result in final mitogenic activity (Cai et al. 2017). IGF1R signaling is also responsible for the maintenance of the transformed phenotype by modulating cancer cell motility, adhesion and angiogenesis (Schips et al. 2004). In our analysis *DOK-1* gene expression has been shown as deregulated upon IGFR signaling which stay in accordance with data of these gene being unfavorable prognostic marker in renal cancer in The Human Protein Atlas. *DOK1* tumor suppressor gene encodes an adapter protein that acts as a negative regulator of several signaling pathways. In particular DOK1 activation inhibits cell proliferation, down regulates MAP kinase activity, has inhibitory effect on leukemogenesis as well as promotes cell motility and apoptosis (Siouda et al. 2014). In terms of RCC specific signaling pathways deregulation it was previously reported that receptor for activated C kinase 1 (RACK1) is a direct mediator between loss of pVHL function and IGF1R signaling in RCC tumor cells. Upon IGF1 stimulation, pVHL-deficient RCC cells exhibit high rate of RACK1/IGF1R binding and up-regulate IGF1R tyrosine kinase activity, with subsequent phosphoinositide 3-kinase/serine-threonine kinase Akt (PI3K/Akt) signaling and matrix metalloproteinase-2 (MMP2) activity resulting in high RCC cells invasiveness (He et al. 2011).

In previous studies clinical significance of IGF-1R was assessed by measuring IGF-1R levels in tissue samples of RCC and non-malignant kidneys. In the analysis of 21 paired specimens higher levels of IGF-1R mRNA were observed in the RCC tumors compared with benign kidney (Yuen et al. 2007). What is more it was showed that antitumor agents - mTOR inhibitors - inadvertently activate the Akt-signaling pathway through an IGF-1R-dependent mechanism (Wan et al. 2007). In the study by Cardillo et al., it was presented that blockade of IGF-1 binding to IGF-1R and downregulation of this receptor are equally effective in inhibiting RCC cell lines growth and combined with mTOR inhibitor – temsirolimus works in synergy. Altogether it suggest new possible approach for treating RCC (Cardillo et al. 2013; Matyszewski et al. 2015a, b, c; Solarek et al. 2015; Tracz et al. 2016). Nevertheless IR and IGF receptors therapies should be developed with caution, as kinase independent biological activities have been described for these receptors (Janku et al. 2013), as well as IGF-2 activation upon IGF-1 blockage (El-Shewy et al. 2007), which seems to be responsible for HEK293 cells viability we report in the presence of inhibitors.

Is this study, we demonstrate that insulin and IGFs are stimulatory factors for RCC cells growth and migration. We also prove that, despite the down-regulation of insulin receptors expression, RCC cells are responsive to insulin stimulation via the IGF1R. In the tumorigenesis process RCC cells do not obtain insulin secretory function and do not activate insulin autocrine stimulation. RCC cell also do not secret significant amount of IGF1 or IGF2, so autocrine stimulation loop in RCC is not responsible for activator effect. As opposed to earlier predictions insulin and IGFs stimulations lead to decrease in expression of many genes of PI3K-Akt-mTOR and Ras-MAPK pathways. These data together are consistent with the conclusion that IGFs and insulin may play a stimulatory role in renal cancer tumorigenesis and progression.

## Electronic supplementary material

ESM 1 (JPG 2670 kb)

ESM 2 (PDF 463 kb)
