# Supplementary figures and images for: Insulin and insulin-like growth factors act as renal cell cancer intratumoral regulators
Source: J Cell Commun Signal. 2019 Mar 30;13(3):381–94. doi: 10.1007/s12079-019-00512-y (PMC6732138; doi:10.1007/s12079-019-00512-y)

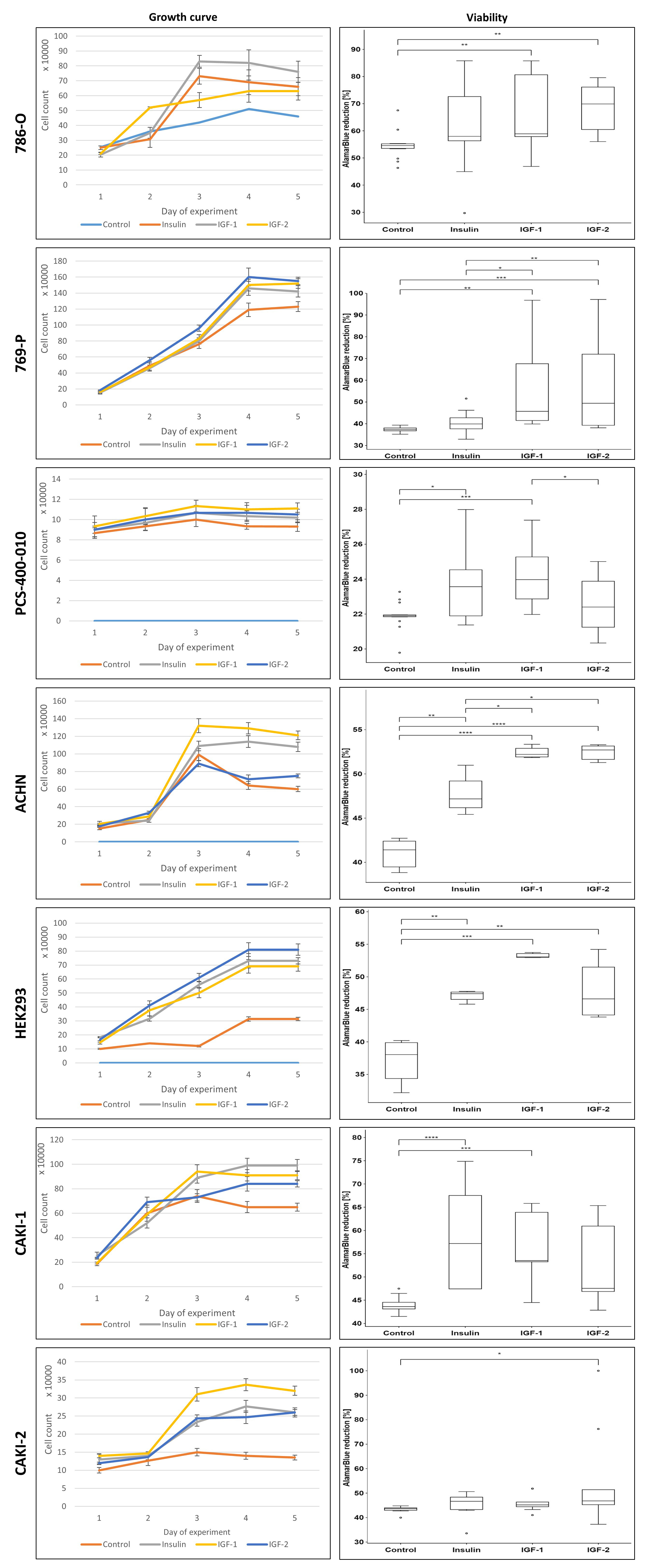

Supplement: Supplementary file 1 — (JPG 2670 kb) [file 12079_2019_512_MOESM1_ESM.jpg]

# 769-P

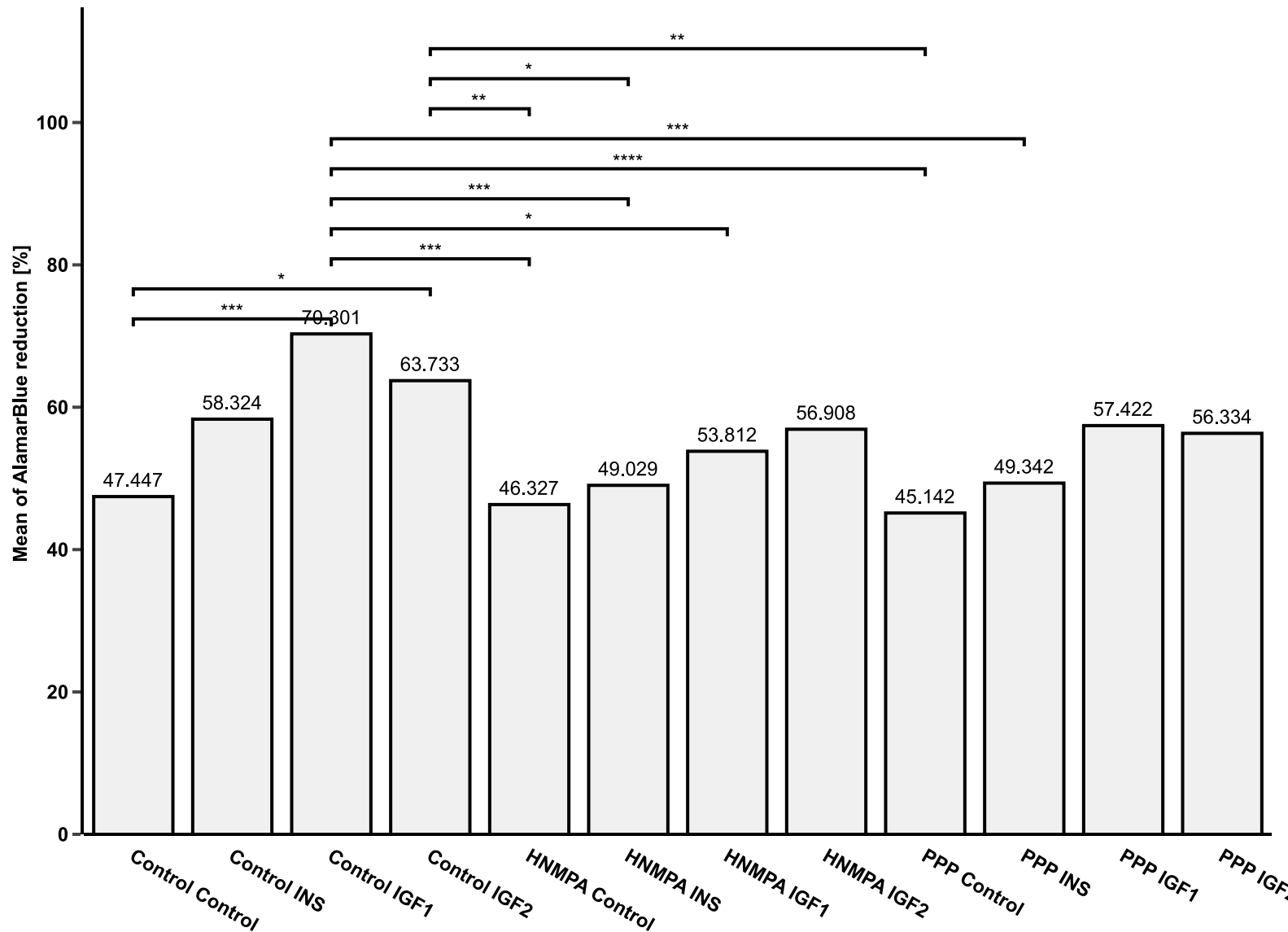

# 786-O

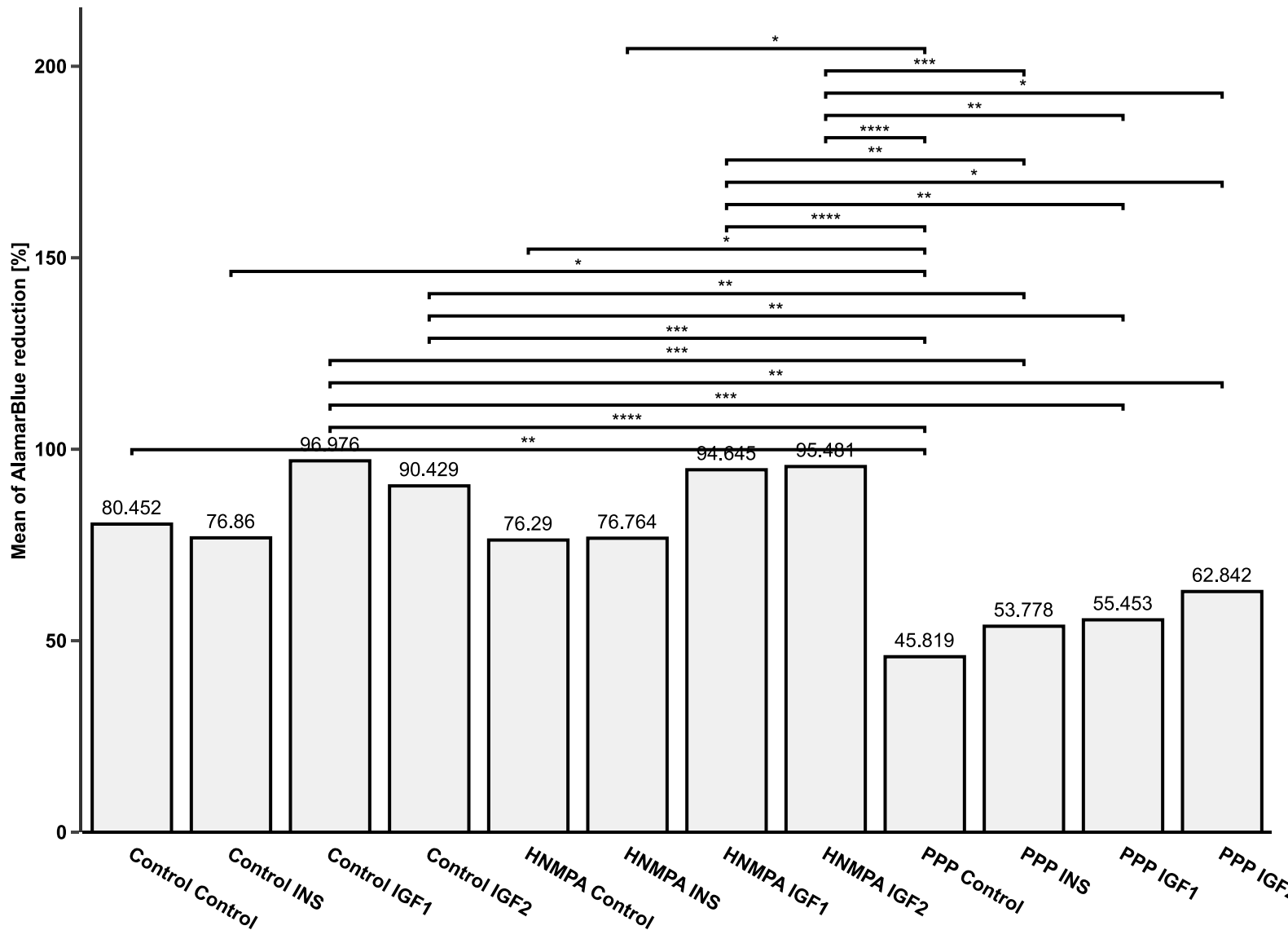

# PCS-400-010

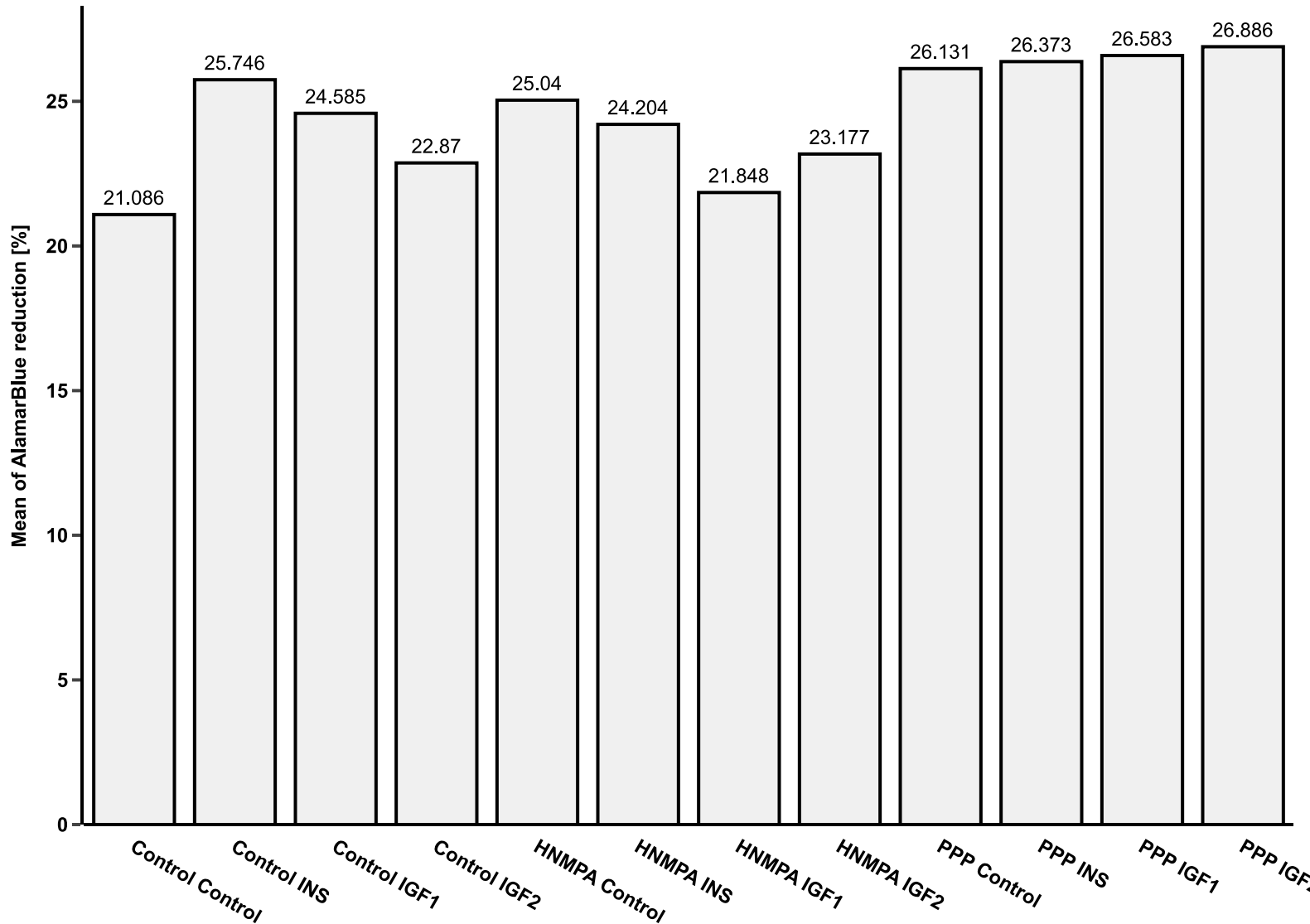

# ACHN

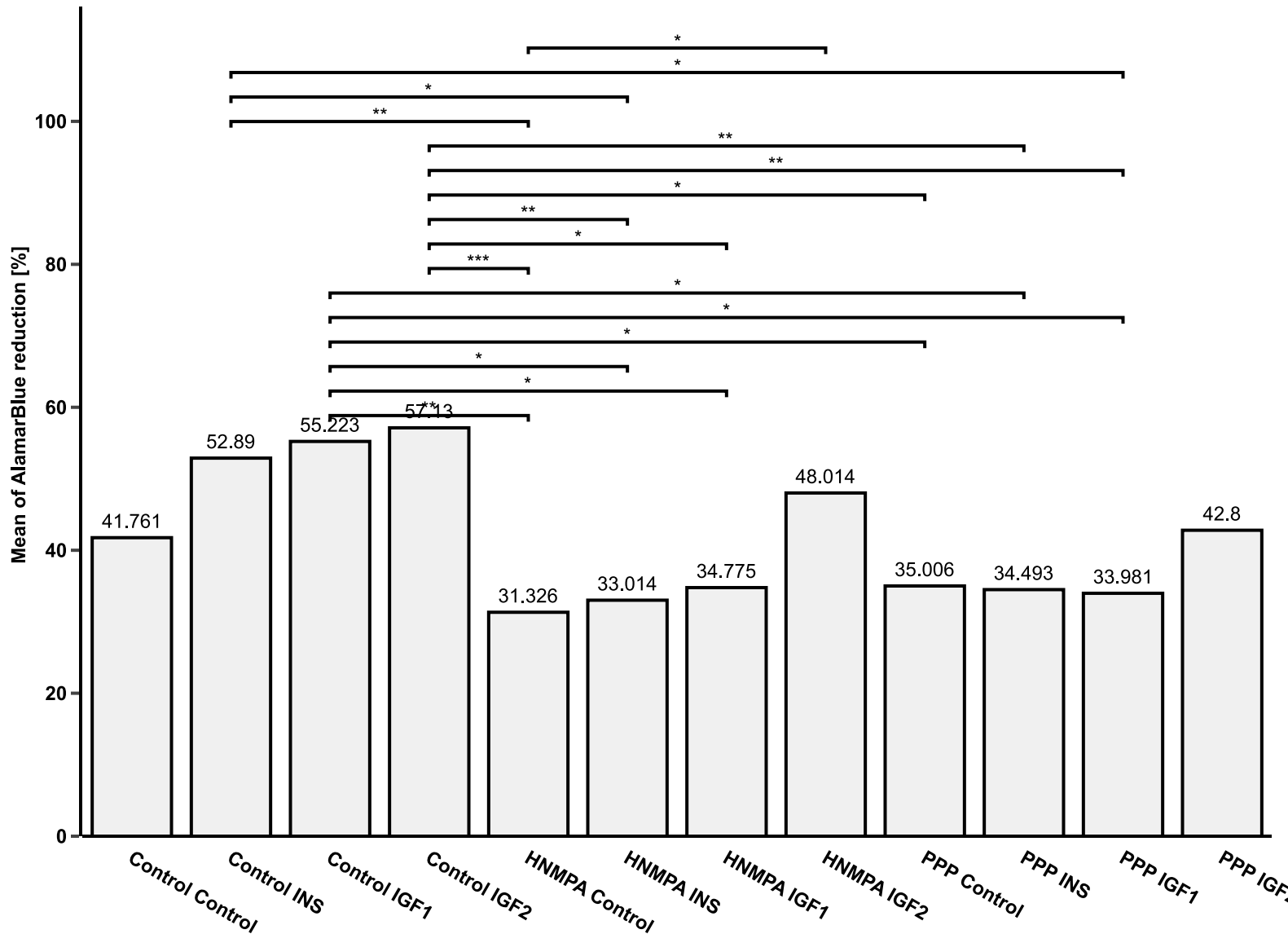

# HEK293

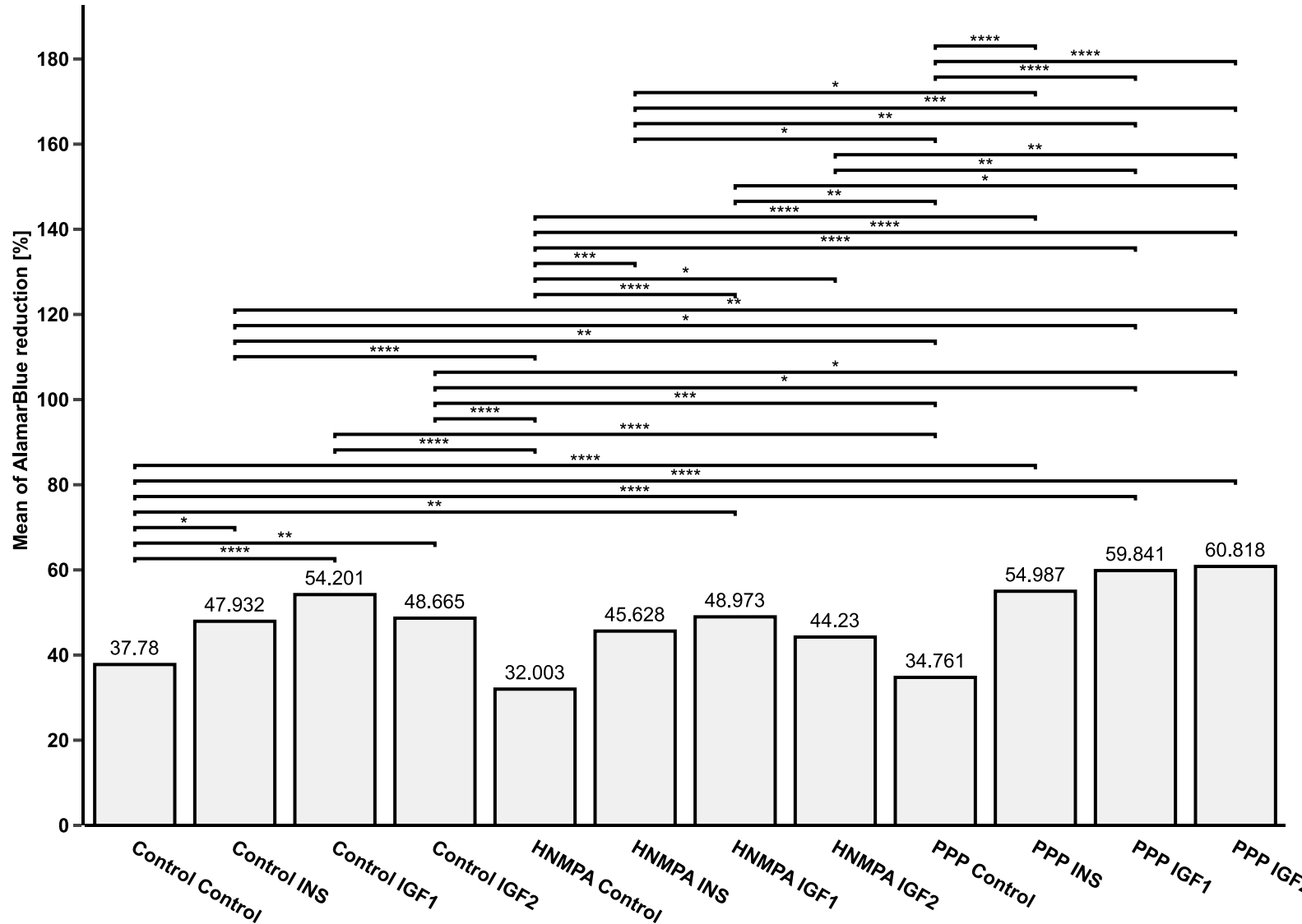

# CAKI-1

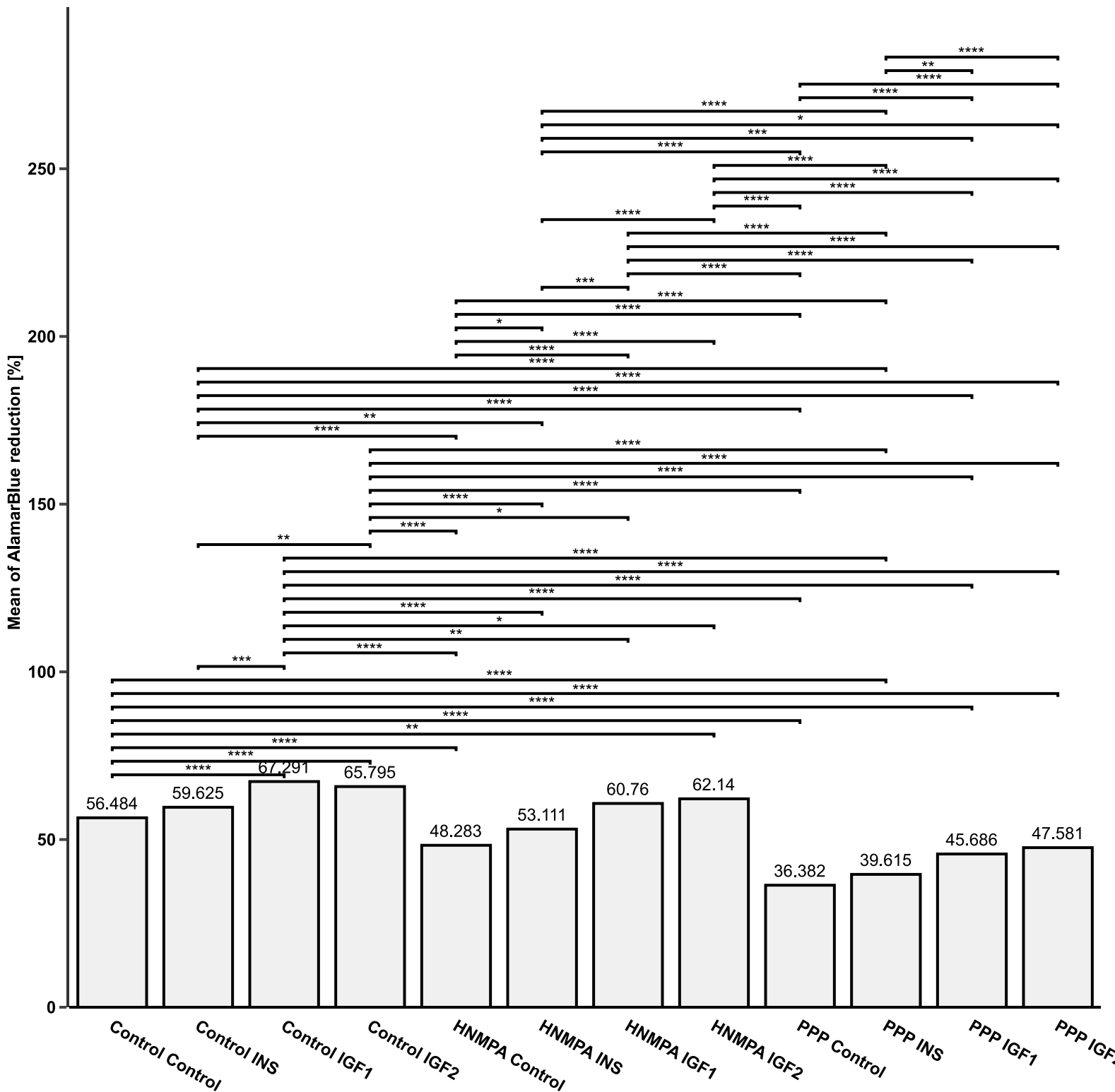

# CAKI-2

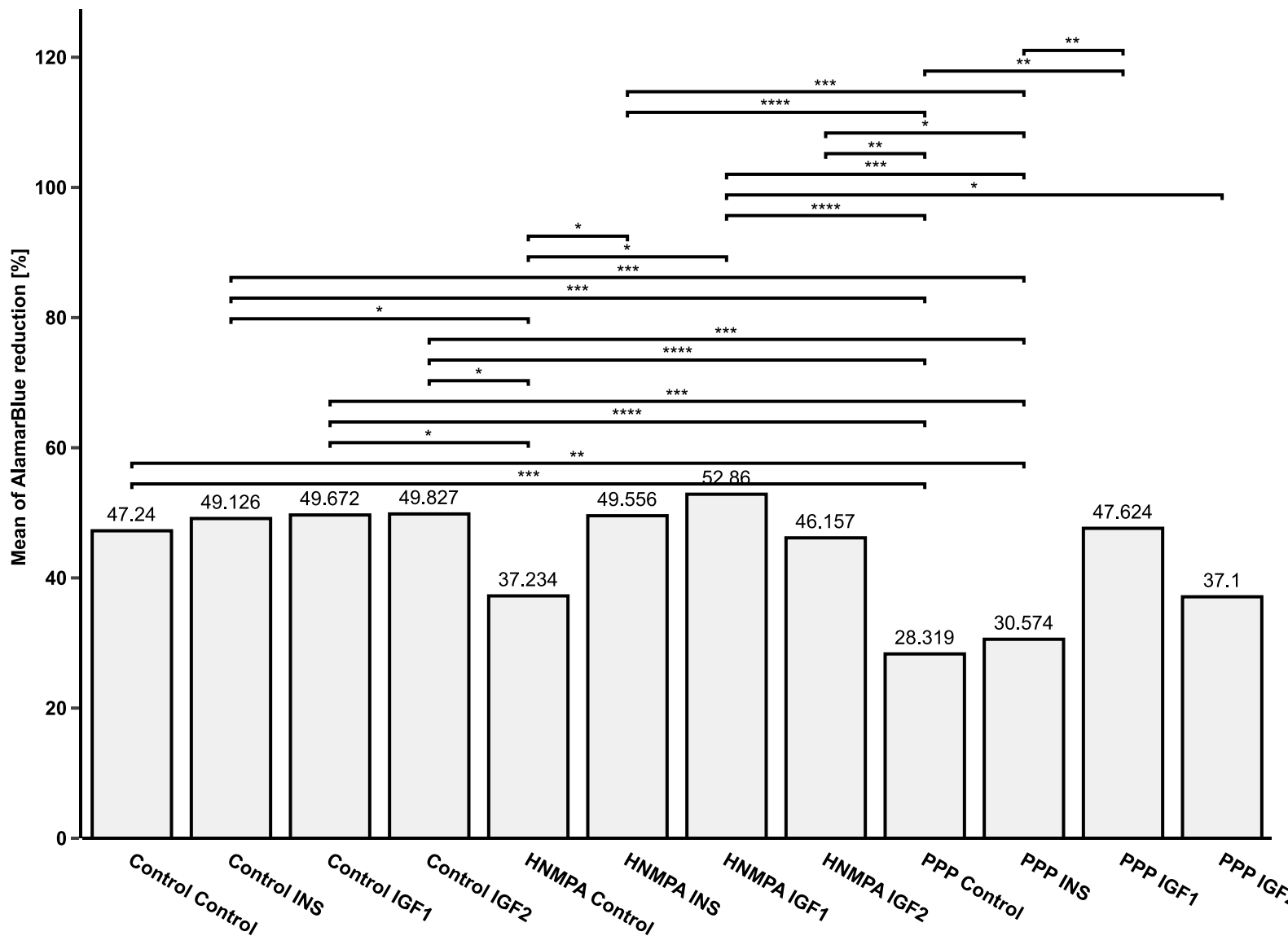

Supplement: Supplementary file 2 — (PDF 463 kb) [file 12079_2019_512_MOESM2_ESM.pdf]
